# Current strategies employed in the manipulation of gene expression for clinical purposes

**DOI:** 10.1186/s12967-022-03747-3

**Published:** 2022-11-18

**Authors:** Hsing-Chuan Tsai, Violena Pietrobon, Maoyu Peng, Suning Wang, Lihong Zhao, Francesco M. Marincola, Qi Cai

**Affiliations:** Kite Pharma Inc, Santa Monica, CA 90404 USA

**Keywords:** Genome-editing nuclease, Transposons, Episomes, siRNA, shRNA, Viral vectors, Non-viral vectors

## Abstract

Abnormal gene expression level or expression of genes containing deleterious mutations are two of the main determinants which lead to genetic disease. To obtain a therapeutic effect and thus to cure genetic diseases, it is crucial to regulate the host’s gene expression and restore it to physiological conditions. With this purpose, several molecular tools have been developed and are currently tested in clinical trials. Genome editing nucleases are a class of molecular tools routinely used in laboratories to rewire host’s gene expression. Genome editing nucleases include different categories of enzymes: meganucleses (MNs), zinc finger nucleases (ZFNs), clustered regularly interspaced short palindromic repeats (CRISPR)- CRISPR associated protein (Cas) and transcription activator-like effector nuclease (TALENs). Transposable elements are also a category of molecular tools which includes different members, for example Sleeping Beauty (SB), PiggyBac (PB), Tol2 and TcBuster. Transposons have been used for genetic studies and can serve as gene delivery tools. Molecular tools to rewire host’s gene expression also include episomes, which are divided into different categories depending on their molecular structure. Finally, RNA interference is commonly used to regulate gene expression through the administration of small interfering RNA (siRNA), short hairpin RNA (shRNA) and bi-functional shRNA molecules. In this review, we will describe the different molecular tools that can be used to regulate gene expression and discuss their potential for clinical applications. These molecular tools are delivered into the host's cells in the form of DNA, RNA or protein using vectors that can be grouped into physical or biochemical categories. In this review we will also illustrate the different types of payloads that can be used, and we will discuss recent developments in viral and non-viral vector technology.

## Gene expression regulation as a strategy to treat genetic diseases

A variety of human diseases are defined by underlying genetic determinants, which may be represented by modifications in gene expression patterns, such as upregulation, downregulation or ectopic expression of wild-type or mutant genes. The physiological expression of genes harboring deleterious mutations may also cause loss-of-function genetic disease. For example, mutations in the *SMN1* gene lead to spinal muscular atrophy type I (SMA 1), the most common genetic cause of infant mortality [1, 2]*.* Gain-of-function mutation is, instead, a type of mutation which confers new or enhanced activity on a gene product, such as for *PIK3CA* E545K. Such mutation results in loss of regulation and constitutive PI3Kα activity, which can lead to oncogenesis [3]*.* Finally, in some instances, dominant negative mutations can occur, creating a protein which adversely affects the wild-type gene product, within the same cell. A classic example is Huntington's disease caused by the expansion of a CAG trinucleotide repeat stretch in the coding sequence of the *HTT* gene [4]*.*

To cure genetic diseases, it is crucial to rewire aberrant host’s gene expression back to physiological conditions. With this purpose, researchers developed molecular tools including genome editing nucleases, transposons, episomes and siRNA/shRNA. Such tools are delivered into the host's cells in the form of DNA, RNA or protein using physical or biochemical methods.

In this review, we describe the different molecular tools that can be used to regulate gene expression and discuss their potential for clinical applications. Moreover, we address the advantages and disadvantages of payload types packed by both viral and non-viral vectors. We focus on recent developments in vector technology and outline the requirements for vectors to succeed in cell and gene therapy.

## Molecular tools to modify gene expression

A variety of molecular tools are available to regulate gene expression. Some of them have been profusely investigated and adapted to clinical use, while others are still being tested at the preclinical stage.

In this section, we discuss different methods to modify host gene expression, through the delivery of genetic payloads. Such strategies include the delivery of genome editing nucleases, transposons, episomes, siRNA and shRNA. Each category presents advantages and disadvantages summarized in Table 1.

**Table 1:** Different systems used to modify gene expression and their main advantages and disadvantages. This is the summary of paragraphs that include references from [1] to [145]

	Options	Key feature	Advantages	Disadvantages
Genome Editing Nucleases	• MNs • ZFNs • TALENs • CRISPR/Cas	• Locus-specific	• One-time treatment • Versatility and multiplex ability	• Off-target effects leading to mutagenesis and translocations
Transposons	• SB • PB • TcBuster • Tol2	• Non locus-specific	• High cloning capacity • Low toxicity • Biosafety	• Transposition efficiency • Insertional mutagenesis
Episomes	• MAC, HAC • Putative ORI • S/MAR family	• Transient (potentially longer)	• No insertional mutagenesis • Remain extrachromosomal • Low toxicity • High cloning capacity	• Gene silencing due to host gene regulation mechanisms • Transfection efficiency • Mitotic instability
Small RNA molecules	• siRNA • shRNA • bifunctional shRNA	• Transient	• No insertional mutagenesis • High efficiency • Chemical modifications to reduce off-target effects	• Transient effect

### Genome editing nucleases

#### Different types of genome-editing nucleases: advantages and disadvantages

Gene editing is a type of genetic engineering that allows the introduction of permanent and locus-specific DNA modifications in the genome. Four types of gene-editing nucleases have been used so far in research: meganucleases (MNs), zinc finger nucleases (ZFNs), transcription activator-like effector nucleases (TALENs) and clustered regularly interspaced short palindromic repeats (CRISPR)-associated endonucleases (Cas) [5, 6].

To guide the nuclease to the target site, MNs, ZFNs, and TALENs use a protein-DNA interaction while CRISPR-Cas systems are guided by RNA–DNA interactions. MNs are highly specific endonucleases, recognizing target sequences of about 14–40 base pairs (bp) [7]. A drawback in using MNs is the limited number of target sites that they recognize and therefore the extreme difficulty to use these endonucleases in clinical settings, where a higher level of flexibility is desirable [8]. The creation of new MNs is a laborious process requiring complex protein engineering procedures because the DNA binding and cleavage domains are difficult to separate [7, 9]. Such laborious engineering required for MNs has constrained their widescale use [10].

The main characteristics of genome-editing nucleases are listed in Table 2. Both ZFNs and TALENs utilize the nuclease FokI as a cleavage domain. Each ZFN module is composed of about 18–36 amino acids and recognizes a specific 3 bp sequence [11, 12]. Therefore, several zinc finger modules need to be engineered to recognize the target sequence: each module will bind 3 bp in the target sequence and the FokI nuclease will be coupled to the DNA-binding modules [13–15]. TALENs follow the same principle, but the DNA-binding module recognizes one single nucleotide, instead of 3 [16]. TALENs modules are composed of 30–40 amino acids, resulting in a protein with higher specificity but larger than ZFNs [17]. Additionally, for both ZFNs and TALENs, it is necessary to engineer two different enzymes for each target, one upstream and one downstream of the cut site. This is necessary because FokI dimerization is required for completion of the double-stranded break. Overall, the engineering of ZFNs and TALENs are technically challenging, and time consuming compared to the engineering of CRISPR-Cas nucleases [8, 18]. Furthermore, the target sequence requirements for ZFNs render the selection of an appropriate and specific target difficult [6, 19, 20].

**Table 2:** Main features of the different genome-editing nucleases

	**ZFNs**	**TALENs**	**CRISPR/Cas**
Phylogenetic origin	artificial restriction enzyme [252, 253]	*Xanthomonas bacteria * [254]	*Streptococcus pyogenes* [255]
DNA binding domain	zinc finger protein [253, 256]	TALE protein [16, 257, 258]	guide RNA [259–261]
DNA cleavage	FokI [262]	FokI [257, 258, 262]	Cas9 [259, 260]
DNA recognition range	18–36 bp (3 bp per module) [253]	30–40 bp (1 bp per module) [257, 258, 263]	22 bp (DNA-RNA base pairing) [261]
DNA cut	dsDNA as a dimer [264]	dsDNA as a dimer [265]	dsDNA complex protein-gRNA [259]
Recognition sequence	5'-GNNGNNGNN-3’ [256]	sequence with 5'-T and A-3' [16, 254, 263]	sequence immediately followed by 5'-NGG-3' [259, 266]
Advantages	Small protein size (< 1 Kb), sequence-based module engineering [267]	High specificity, easy selection of target region [268]	Easy to multiplex, simple synthesis of gRNA, easy selection of target region [269]
Disadvantages	Difficult sequence selection and protein engineering,, expensive and time consuming [267]	Large protein size (> 3 Kb), expensive and time-consuming [269, 270]	Large protein size (> 4 Kb) [269]
Safety concerns	off-target effects: genome mutagenesis and GCRs [271]	off-target effects: genome mutagenesis and GCRs [270]	off-target effects: genome mutagenesis and GCRs [271]

#### CRISPR-Cas technology

CRISPR-Cas nucleases have crucial advantages compared with ZFNs and TALENs, including the simplicity of the guide RNA (gRNA) design (Table 2). Such nucleases use a 22 bp gRNA to bind a complementary target sequence, which is subsequently cut by the Cas itself [8, 21, 22]. By designing specific gRNAs, CRISPR-Cas systems could theoretically target any sequence in the genome. Indeed, the ease of target selection and the possibility of multiplexing the gRNAs while maintaining high specificity and efficiency led to the rapid development of CRISPR-Cas methods for clinical purposes [18]. CRISPR-Cas systems allow to rapidly screen a large number of gRNAs and the scalability of this platform permits an accurate optimization of the study system [23–25].

Stable integration of CRISPR-Cas is not necessary to provide a therapeutic effect and long-term expression is usually considered a disadvantage, as it can lead to off-target cleavage. However, the persistence of CRISPR-Cas in the cell must be sufficient to perform the editing function [26].

The delivery of a native Cas protein in complex with a gRNA bypasses the requirement for transcription and translation. It introduces genome editing approximately 3 h after delivery and is degraded after 24–48 h [26]. Circumventing transcription and translation is useful in post-mitotic or hard-to-transfect cells. Such transient functionality allows for rapid editing and reduced off-target effects. However, obtaining pure active protein is a difficult process, and the risk of endotoxin contamination remains of concern [27]. The delivery of Cas proteins offers an improved dose-control compared to mRNA and DNA but, in order to produce a therapeutic effect, a significant amount of protein must be successfully delivered. This is due to the lack of amplification signal which normally occurs with mRNA and DNA. Moreover, the CRISPR-Cas protein is large, which may present a challenge for intracellular delivery.

Another strategy to minimize off-target editing events is through the delivery of Cas mRNA. This process results in rapid genome editing (5–7 h after transfection) and avoids the step of nuclear entry [28]. The mRNA that codes for the Cas protein is immediately translated in the cytosol and the complex Cas-gRNA subsequently enters the nucleus. The transient expression of Cas proteins reduces off-target effects and risk of integration, but the dose and timing of mRNA delivery have to be carefully titrated [29, 30].

Finally, the delivery of genome editing nucleases through plasmids is an easy procedure. Due to the necessity of nuclear entry, subsequent transcription, and translation into protein, the genome editing efficacy is significantly delayed. Plasmids are very stable molecules, therefore Cas protein expression may last several days, leading to a high risk of off-target effects and safety concerns [31, 32]. Plasmid delivery may also trigger cytosolic DNA toxicity [33]. Despite the fact that each of the above-mentioned nucleases present specific advantages and disadvantages, CRISPR-Cas technology has been widely adopted and improved in the last few years and it remains a promising route for preclinical and clinical investigations [8]. Depending on the cell type and experimental conditions, the knockout efficiency for CRISPR-Cas9 varies between 40% (induced pluripotent stem cells, iPSCs) and 99.4% (cortex, hippocampus and spinal cord) [34–37].

CRISPR-Cas9 can also be used for gene or targeted nucleotide knock-in experiments. Such manipulations are usually more challenging to perform and require accurate optimization, including the addition of an extra component in the form of a DNA donor template [38]. Different studies reported a wide range of knock-in efficiencies depending on the method and the cell type used. Liu et al. [39] compared the efficiencies of CRISPR-Cas9 versus ZFN and TALEN, performing knock-ins in fetal fibroblasts [39]. They found that CRISPR-Cas9-mediated gene knock-in (70–80% efficiency) was 5.6 times more efficient than ZFN and around 3 times more efficient than TALEN. Nevertheless, other studies found lower knock-in efficiencies for CRISPR-Cas9, for example ∼20% in human primary T cells [40]. In conclusion, the overall efficiency of knock-in seems to remain lower than the knockout efficiency, using CRISPR-Cas9. Moreover, delivery of CRISPR-Cas9 through viral vectors requires in vitro T cell activation and culture [41, 42]. However, the use of electroporation (EP) methods to deliver Cas9-gRNA protein complex in knockout studies demonstrated the potential to overcome this issue and to achieve gene editing without in vitro T cell activation [42, 43]. Human primary T cells are difficult to manipulate and chemically modified gRNAs were also tested to enhance genome editing efficiency [44].

In the last decade many tools have been developed and optimized to investigate genome functional complexity based on Cas proteins. Among those, a Cas-based tool for epigenome editing (non-gene editing) called “nuclease dead Cas” (dCas) was developed by creating a mutant form of Cas which lacks endonuclease activity. This enzyme still retains the capability to bind the gRNA and it can target Cas-coupled effector proteins to a specific locus of the genome [45]. Coupling dCas with VPR activator (CRISPRa) or KRAB repressor (CRISPRi) of transcription creates a powerful tool for precise epigenetic editing. For example, Schmidt et al. [46] developed a CRISPRa and CRISPRi platform to perform genome-wide screens for functional regulators of cytokine production in response to T cell stimulation [46]. Yang et al. [47] developed a CAR-T cell product called RB-340–1, which was engineered through a CRISPRi circuit to prevent Programmed cell Death protein 1 (PD-1) expression upon antigen-encounter [47]. RB-340–1 is the first application of CRISPRi toward a clinically relevant product and allows the conditional and reversible suppression of PD-1. The reversible nature of this editing also allows fine tuning of the degree of PD-1 expression. RB-340–1 demonstrated resilience to checkpoint inhibition and increased persistence and effectiveness against HER2-expressing cancer xenografts [47].

#### Safety of genome editing-based techniques

A drawback that should be considered when performing genome editing is the immunogenicity of the nucleases – ZFNs, TALENs and CRISPR-Cas are exogenous proteins and may trigger an immune response in the patient. Regarding SpCas9, in vivo delivery has been found to elicit both antibody and T cell responses in immunocompetent mice [48–51]. Cell therapy which employs products transiently edited ex vivo through plasmids, mRNA or protein, is expected to be safe as the Cas9 is diluted during cell proliferation [52]. Early reports from clinical trials revealed persistence of T cells edited ex vivo through SpCas9, in cancer patients [53–55]. However, in all these reports Cas9-directed immune responses were not directly evaluated [56] and the available safety data derived from patients that had a compromised immune system. Despite the encouraging results, thorough investigations with ex vivo engineered T cell products may be needed to assess humoral and cellular immune response, after infusion.

Off-target genotoxicity together with the risk of creating translocations when multiplex genome editing is performed are a major drawback of genome editing nucleases [57]. Indeed, chromosomal translocations are natural byproducts of inducing simultaneous genomic breaks [58, 59]. Different nuclease combinations or the presence of a homologous single-stranded donor have been suggested as approaches to reduce chromosomal translocations in multiplex editing [58]. For example, Bothmer et al. [58], performed knockout at the TRAC and B2M loci in human T cells, including a single-stranded repair template in the reaction. The repair template presented 70 bp of homology on either side of the double-strand break (DSB) with a 10 bp stop cassette, to achieve functional knockout. With this strategy, the DSB repair mechanism was shifted from Non-Homologous End Joining (NHEJ) which can cause translocations, to single-stranded template repair (SSTR) [58].

#### Clinical trials

Over the past few years, genome editing nucleases made their appearance in clinical trials although so far, no U.S. Food and Drug Administration (FDA) approved treatment based on this technology has been commercialized and no late-stage clinical trial has been approved.

In gene therapy, ZFN and CRISPR-Cas9 are currently being investigated in clinical trials to treat genetic diseases such as Mucopolysaccharidosis (NCT03041324, NCT04628871, NCT02702115), Hemophilia B (NCT02695160), β-Thalassemia (NCT03432364, NCT03728322, NCT03655678), Neurofibromatosis type 1 (NCT03332030), Sickle cell disease (NCT03745287) and LCA10 (NCT03872479). All these clinical trials are at an early stage: phase I or I/II and no clinical trials have been approved for TALEN so far.

The situation is similar for cell therapy: early-stage clinical trials are currently evaluating products to treat both hematological malignancies and solid tumors. Genome editing nucleases are employed to knock out target genes such as IL13Ralpha2, PD-1, CISH, TRAC and B2M. For example, TALEN is currently being tested in a clinical trial to knockout TRAC and CD52 in allogeneic CAR-T cells (NCT02808442). This is to create an off the shelf CAR product for a specific patient population [60]. ZFN has been used to permanently disrupt the glucocorticoid receptor GRm13Z40-2 in anti-IL13Ralpha2 allogeneic CD8 + T cells, used to treat patients with recurrent/refractory malignant glioma (NCT01082926). Products engineered through CRISPR-Cas9 technology have been more frequently adopted in clinical trials. For solid tumors, PDC1 and TRAC knockouts have been tested (NCT02793856, NCT03081715, NCT03044743, NCT03545815, NCT03747965) aiming to decrease CAR-T cells exhaustion. For hematological malignancies CRISPR-Cas9 target genes include TRAC, B2M, CD7, CD28, CD19, CD20 and CD22 (NCT03190278, NCT03166878, NCT03398967, NCT03690011), depending on the cancer characteristics.

### Transposons

Transposable elements (TEs), also known as transposons, are sequences of DNA that move from one location to another in the genome. TEs have been identified in all organisms and can comprise a large proportion of a species’ genome [61, 62]. Class I retrotransposon replicates by a copy-and-paste mechanism, producing intermediate mRNA copies that are reverse transcribed [63]. Class I retrotransposons make up 40–50% of the human genome and include long and short interspersed repeats (LINEs and SINEs) and long terminal repeat elements (LTR) [61]. Class II DNA transposons which are not active in humans, utilize a cut-and-paste mechanism to move DNA elements from one location to another [64, 65]. In some cases, such as *Helitrons*, a peel-and-paste mechanism, involving a circular DNA intermediate, is used to move and replicate DNA elements in the genome [66].

DNA transposons consist of a transposase gene that is flanked at both ends by terminal inverted repeats (TIRs) [67]. The expressed transposase recognizes both ends of TIRs and deploys cut-and-paste mechanisms to move the entire transposon to another location of the genome [68]. Taking advantage of TIR sequences and transposase activity, different types of transposons have been used for genetic studies and serve as gene delivery tools (Table 1). In the design of delivery constructs, the gene of interest (expression cassette) is flanked by TIRs on both ends, and the transposase gene can be delivered separately via mRNA, protein, or DNA.

#### Different types of transposons

Different types of TEs have been studied, the most common being used as genetic tools in mammalian cells, including Sleeping Beauty (SB), PiggyBac (PB), TcBuster, and Tol2 [67]. All are mobilized through a cut-and-paste mechanism [69–71]. TEs are active in various species from protozoa to vertebrates, including mice, rats, and humans. Several studies demonstrated that in mammalian cells, PB and SB have high transposition activity, with PB having stronger activity [72–74].

Most natural DNA transposons are inactive during evolution for genome stability [72]. Hyperactive transposases have been designed through rational amino acid substitution and codon optimization to increase the transposition efficiency*.* For example, the hyperactive SB100X and SB150X TEs possess respectively 100-fold and 130-fold higher transposition activity compared to the original SB10 [75–77]. Hyperactive SBs have been used in germline transgenesis in rodents, rabbits, and pigs, and to reprogram mouse embryonic and human fibroblasts into iPSCs [78–85]. The wild-type PB (PBase) was codon-optimized for mammalians (mPBase) demonstrating a 20-fold increase in transposition efficiency, compared to PBase [86]. Subsequently, seven amino acid substitutions were combined, leading to the hyperactive PB called hyPBase [87]. hyPBase demonstrated a 17-fold increase in excision and a ninefold increase in integration, compared to mPBase. Recently, a new hyperactive variant of TcBuster has been commercialized and has been used in a variety of studies [88]. A final example is the 6X-His-tagged variant of Tol2 transposase, which maintains high transposition activity in vitro and in vivo. Such a tag-targeted modification of Tol2 allowed the identification and purification of the Tol2 transposase from cells, a process which is useful for biochemical studies [89]. This is an important result because targeted modification of transposase enzymes often results in reduced enzymatic activity, as was the case for SB [74, 90].

During the past few decades, variants of TIRs such as truncations and changes in their configuration were also generated to improve transposition efficiency [91]. The truncation of PB’s TIR led to the development of IRmicro, which increased the transgene expression in vivo by 1.5-fold [92]. Over the years different miniTol2 were created as well. However, in these studies, the minimal TIR sequence did not improve transposition activity in vivo and in vitro compared to the original TIR [89, 93, 94].

Regarding SB, each TIR unit on both ends is 200–250 bp in length. The left TIR also contains an extra half direct repeat (DR) to enhance element transposition. Modification of the DRs and the space sequence between the DRs lead to the development of pT2, which displayed a fourfold increase in transposition efficiency compared to the first-generation transposon [95]. In the sandwich transposon (SA), the TIR elements consist of two complete transposon elements in a head-to-head configuration, which flank a DNA expression cassette. The SA displayed a 3.7-fold increase in transposition compared to SB10 [96]. The pT3 vector contains a duplication of the left TIR, which acts as a transposition enhancer. Moreover, it has an extra TA dinucleotide flanking the transgene, to promote increased excision. pT3 showed a threefold increase in efficacy in vivo [97]. pT3 showed a threefold increase in efficacy in vivo. The DNA-recognition domain of the transposase contains two subdomains: PAI and RED. The pT4 vector was created by introducing mutations in the PAI interaction motifs of the inner DRs. The new construct showed a twofold increase in activity compared to pT2 [98].

#### Safety of transposon-based techniques

The members of the Tc1/mariner transposon family, such as SB, present ‘overproduction inhibition’, which refers to loss of transpositional activity in the presence of increasing concentrations of transposase [99]. This negative dosage effect was observed in vitro and in vivo [100] and may be beneficial in clinical settings in terms of biosafety. Data regarding PiggyBac is contradictory, as different studies have inconsistently detected overproduction inhibition [74, 101, 102]. Tol2 and TcBuster transposition is directly proportional to the level of transposase, and these systems lack overproduction inhibition [102, 103].

Since TEs are also randomly integrated in the host genome, like retroviral integration, there is potential insertional mutagenesis risk. Transposon-associated CRISPR-Cas delivery systems have also been developed, using RNA-guided DNA transposition to target the integration of transgenes into specific sites, which would provide further biosafety advantages [104–106].

So far, no evidence has been provided of transposon-mediated transgene integration inducing gross chromosomal rearrangements (GCRs). However, studies have reported cases of rearrangements when a pair of transposition-competent elements is present at the same locus [107, 108]. Remobilization of transposons in the genome may also result in GCRs [109]. More investigations need to be carried to understand the risk.

Transposons that utilize the cut-and-paste mechanism exhibit an effect called ‘local hopping’ when mobilized from a chromosome. Local hopping refers to the preference for cis-integration into sites that are in proximity of the donor locus [110]. Therefore, local hopping confines the target region which is available to a transposon moving from a donor locus. The extent of this phenomenon varies among different TE types, species, and the donor locus itself. SB and Tol2 exhibits local hopping [111, 112]. PB, despite presenting a certain degree of local hopping, has a wider integration range [73, 113, 114]. Local hopping may be a double-edged sword, depending on where the TE is located. If such a region is rich in coding sequences, local hopping might increase the risk of gene mutagenesis.

In conclusion, transposons may be a potential technology to achieve long-term expression of transgenes and may be used in clinical settings. A robust study and construct design are still needed to further validate and avoid undesirable effects in patients.

#### Clinical trials

One Phase I/II clinical trial (NCT04284254) has been approved, testing the use of SB to treat patients with Hurler syndrome [115]. No clinical trials for PB have been approved in gene therapy. Regarding adoptive cell therapy, the landscape is more dynamic with a variety of active clinical trials for both SB and PB. Currently, SB and PB are being investigated to deliver CD19, BCMA, SLAMF7, CD33 or CD116 CAR for immunotherapy of hematological malignancies (NCT03389035, NCT04499339, NCT03927261, NCT00968760, NCT01497184, NCT03288493, NCT04960579, jRCT2033210029, ACTRN1261700157938, ChiCTR1800018111). All these trials are in Phase I/II.

### Episomes

Episomes are defined as closed circular DNA molecules which are autonomously replicating. Episomal vectors may be an interesting clinical strategy due to their long-term expression and intrinsic characteristic of extrachromosomal maintenance, which prevents insertional mutagenesis (Table 1). With proper design and modification, episomes also provide large payload capacity and reduced toxicity [116, 117]. This applies in particular to episomal minicircles (MCs), which harbor reduced amounts of bacterial sequences. However, episomal vectors present some disadvantages, including the possibility of transgene silencing due to epigenetic modifications of the vector [118]. Indeed, episomal genes remain subject to host gene control mechanisms and may undergo modifications such as methylation or deacetylation. Therefore, reducing bacterial sequence and CpG contents may improve transgene expression. Another drawback is their mitotic instability, referred to as episome loss after multiple cycles of cellular division. Proper designs of episomal vectors can be carried to improve mitotic stability, such as pEPI-1 with an S/MAR sequence [119].

#### Different types of episomes

Episomal vectors are divided into three main categories: (A) mammalian or human artificial chromosomes (MAC, HAC), (B) episomes including putative origins of replication (ORI) and (C) episomes including chromosomal scaffold/matrix attachment region sequences (S/MAR) [116]. MAC or HAC possess the highest cloning capacity and harbor telomeric and centromeric sequences to ensure mitotic stability.

Several efforts have been made to create episomes including putative ORI, for use in mammalian cells and only a small number of attempts have been successful because mammalian ORI do not share sequence homologies [120, 121].

S/MAR are genomic DNA sequences that mediate structural organization of the chromatin, anchoring the chromatin to the nuclear matrix protein SAF-A during interphase and thus facilitating DNA segregation into dividing cells [122, 123]. Therefore, the S/MAR family of episomes presents increased mitotic stability compared to conventional plasmids. A variety of S/MAR episomes have been developed over the years, including pEPI-1, PEPito, pEPI-cHS4, pEPI-UCOE, pEPI-TetON, pEdER-IR and pEP-IR [122, 124–128]. To improve vector efficacy, S/MAR MCs were created lacking the bacterial backbone, antibiotic resistance sequences, and bacterial origins of replication [116, 129, 130]. Minicircles have been successfully used in preclinical studies in numerous cell types including CAR-T cells [131–134]. Recently, a new system called nano-S/MAR (nanovector) has been developed [135]. Nanovectors are easier to produce compared to MCs, with a final product of higher purity. Nano-S/MAR vectors have shown improved efficiency of establishment, transgene expression, and minimal cytotoxicity in preclinical studies [135, 136]. Despite the successful application of S/MAR vectors in vitro and in vivo*,* no clinical trial involving their use has been approved so far.

### siRNA and shRNA

RNA interference (RNAi) is also known as RNA silencing or post-transcriptional gene silencing (PTGS) and it is mediated by three classes of molecules: siRNA, shRNA and bifunctional shRNA (Table 1). RNAi is a strategy to transiently regulate gene expression in a wide range of eukaryotes, without permanently modifying nuclear DNA [137].

SiRNA is a class of double-stranded RNA molecules of typically 20–24 base pairs in length. After being delivered to the cell, the siRNA molecule is incorporated into the RNA-Induced Silencing Complex (RISC) where it is unwounded to a single strand RNA. The less thermodynamically stable RNA strand is then used by RISC to probe and anneal with target complementary mRNA. The target mRNA is subsequently cleaved. Alternatively, the siRNA molecule can be incorporated into an RNA-induced transcriptional silencing (RITS) complex, which triggers heterochromatin formation at the locus where the siRNA binds its complementary sequence of DNA. Allele-specific siRNAs (ASP-siRNA) have also been developed to minimize off-target toxicity and selectively inhibit the mutant allele of a gene [138]. This is possible thanks to their ability to distinguish the target from the wild-type sequence, with single-nucleotide specificity. ASP-siRNAs have the potential to be employed in vivo for gene therapy, to silence the dominant mutant allele and to treat a variety of genetic disorders [138, 139].

SiRNA molecules produce a transient effect, especially in rapidly proliferating cells and this characteristic may be considered as a double-edged sword. In the situation where a more durable effect is desired, the siRNA sequence is modified into a shRNA. ShRNAs contain a tight hairpin turn and are encoded in a DNA vector to be delivered into cells. A shRNA vector can provide a more long-lasting effect than just treating cells with siRNA, as it allows for a durable expression of shRNA. Expression of shRNA may also be controlled through inducible or tissue-specific promoters. ShRNAs are transcribed in the nucleus and then processed by Drosha/DGCR8 complex and Dicer/TRBP/PACT into mature shRNA. Mature shRNAs are loaded on Argonaute protein containing RISC complex and thus provide RNAi activity. Finally, bi-functional shRNAs have been developed to obtain a more rapid and efficient RNAi function [137]. These RNAs can be associated with cleavage-dependent and –independent RISCs, simultaneously inducing target mRNA degradation and inhibiting translation through mRNA sequestration.

#### Clinical trials

In 2018 the first U.S. siRNA product was approved by the FDA. Onpattro^®^ (patisiran) is a siRNA product aiming to treat the rare hereditary disease transthyretin-mediated amyloidosis, in adult patients. The majority of siRNA products are intended to treat cancer and orphan/rare genetic diseases. Other Alnylam’s commercially available products are Givlaari^®^ (givosiran) for acute hepatic porphyrias and Oxlumo^®^ (lumasiran) as the first treatment for primary hyperoxaluria type 1. Leqvio^®^ (inclisiran) was FDA-approved in December 2021 and is a treatment to lower cholesterol for people with atherosclerotic cardiovascular disease. The most recently approved siRNA is Amvuttra™ (vutisiran) for treatment of the polyneuropathy of hereditary transthyretin-mediated amyloidosis (June 2022).

A product recently tested in Phase 3 clinical trials is Fitusiran (NCT03417245). Data from Phase 3 demonstrated that Fitusiran significantly inhibits bleeding in patients with hemophilia A or B [140]. Another advanced clinical trial is currently testing Teprasiran (Phase 3—NCT03510897), a siRNA which inhibits p53-mediated cell death that underlies acute kidney injury (AKI) in high-risk patients undergoing cardiac surgery. The incidence, severity and duration of AKI were significantly reduced after Teprasiran administration [141]. Cosdosiran (Phase 2/3—NCT02341560) is being evaluated to improve visual function in subjects with recent-onset acute nonarteritic anterior ischemic optic neuropathy (NAION). Lastly, Nedosiran (Phase 2—NCT03847909) is an siRNA that inhibits hepatic lactate dehydrogenase in patients with primary hyperoxaluria and Tivanisiran (Phase 3—NCT04819269) is a therapy for dry eye disease due to Sjögren's Syndrome [142].

Some early-stage clinical trials adopt a combo approach using siRNA and immune cells. In order to knock down the CD3ζ component of the TCR, shRNA was tested in a new allogeneic product (CYAD-211) that co-express an anti-BCMA CAR (NCT04613557). The product was well-tolerated and two partial responses were observed among five evaluable patients [143]. Finally, in the clinical trial NCT03208556, iPD1 CD19 eCAR T cells were tested in relapsed or refractory B-cell lymphoma. A PD1 shRNA-expressing cassette was incorporated in the CAR lentivector with the objective to improve anti-tumor activity by inhibiting PD1 induction.

## Type of payloads to deliver molecular tools for gene expression regulation

The molecular tools described above can be delivered into cells using various forms of genetic materials (such as DNA, RNA) and protein, based on the intended purpose (e.g., transient or long-term expression), the different genetic tool design and the delivery methods.

### DNA-based payload

Plasmid DNA (pDNA) is the most widely used payload in gene and cell therapy. pDNA is usually composed of a supercoiled double-stranded DNA of variable size (< 1 kb to 200 kb). Typically, pDNA contains an antibiotic resistance gene, a prokaryotic origin of replication for plasmid propagation, and an expression cassette [144, 145]. The cassette is usually composed of a gene of interest, a promoter for its transcription, and a polyadenylation signal for mRNA export. However, the large backbone and antibiotic resistance gene limit their use in gene and cell therapy due to reduced biocompatibility and safety [146]. The unmethylated CpG motif contained in a variety of plasmids has been shown to induce an immune response via TLR9 signaling and may trigger inflammation and tissue damage when administered in vivo [147]. In addition, bacterial sequences in the plasmid can contribute to transgene silencing in host cells. Moreover, the size of the plasmid can significantly impact transfection efficiency. Efforts have been made to downsize the bacterial fragments and antibiotic resistance genes to improve biocompatibility, durability, and safety [148].

Other forms of DNA payload have also been explored for cell therapy. Linear DNA synthesized through PCR has entered the cell therapy arena to challenge the traditional use of bacterial plasmid DNA, however linear DNA also possesses the risk of foreign DNA and endotoxin contamination. Linear DNA can be used in combination with other payloads in EP. Roth et al. [149] demonstrated that electroporation of primary T cells with CRISPR-Cas9 RNPs and linear double-stranded DNA achieved a transfection efficiency of ~ 50% as shown by the GFP reporter signal [149]. As mentioned before, minicircles are supercoiled DNA molecules with very few bacterial sequences [130]. Improvements in transfection efficiency and prolonged expression have been reported for MCs and they are considered having a favorable safety profile. Other DNA forms, such as DNA ministrings [150] and nano/mini-plasmids, have also shown improvements in transfection efficiency by reducing the unnecessary bacterial sequences.

Ministring DNA is a plasmid-derived DNA delivery system which possess linear covalently closed (LCC) ends, DNA targeting sequences at both ends and transgene expression cassette devoid of prokaryotic sequences [150]. Mini and nanoplasmids are small circular DNA constructs and do not possess antibiotic resistance genes [151].

Gene delivery systems should consider the toxicity associated with DNA delivery, which includes the sensing of cytosolic DNA by host cells [152, 153]. Cellular DNA sensors detect cytosolic genetic material, usually due to viral infections or self-DNA leaking from different cellular compartments [154–157]. The detection of cytosolic nucleic acids triggers the host immune response and is pivotal for mammalian organisms to control malignant transformation and to mediate cell-intrinsic onco-suppression [158]. The accumulation of cytoplasmic DNA can lead to cellular senescence or regulated cell death through multiple pathways, involving proteins including STING, ZBP1, AIM2 and IFI16 and therefore creating vector dependent DNA-associated cytotoxicity [159–163].

### RNA-based payload

Small mRNA payloads are preferred to larger pDNA regarding post-transfection cell viability. However, there are innate limitations for mRNA-based gene editing. For example, in vitro-transcribed (IVT) mRNA is less stable than DNA. This problem could be mitigated by introducing chemical modifications in the synthetic RNA, such as adding a 5’-methylated cap and poly(A) tail (reviewed in[164]). Similar to DNA, mRNA also elicits innate immune response. The strategy consists of employing modified nucleosides, including pseudouridine, N1-methylpseudouridine, 2-thiouridine, 5-methylcytidine, or N6-methyladenosine to dampen the immunogenicity of the mRNA [165, 166]. Thanks to these improvements, IVT mRNA has been used in CAR-T cell studies on hematological and solid tumors, some of which have advanced to clinical trials (reviewed in [167]).

Another concern is durability: since mRNA does not integrate into the genome, the transgene expression is usually transient and may require multiple doses for long-term expression. Multiple studies showed that EP of mRNA results in CAR expression that lasts for around seven days [168–170], potentially reducing on-target-off-tumor toxicity [171]. It may also be useful to target antigens with specific spatial and temporal patterns, as in the recent example where mRNA engineered CAR-T cells were used to treat cardiac injury [172]. Finally, small regulatory RNAs (microRNA, shRNA, siRNA, etc.) are used in gene and cell therapy to transiently silence a target gene, as discussed above. This is a targeted approach that has been investigated for the treatment of cardiovascular diseases, viral infections, and cancer. Researchers are currently attempting to address the drawbacks of small regulatory RNAs such as stability, extracellular and intracellular barriers, and innate immune stimulation (reviewed in [173–177]).

### Protein-based payload

Most macromolecules such as proteins do not enter cells by passive diffusion because of their limited intrinsic capability to internalize. Membrane disruption and cell-penetrating peptides have been used for cross-membrane transport of large molecules. Payload proteins include antibodies, transcription factors, and genome editing nucleases [178]. Most notably, proteins used in cell therapies are usually components of gene-editing nucleases and transposons. The advantage of using protein payload is to precisely control the dose and expression time frame of interest genes. Proteins could also be delivered by nanoscale injection or localized EP devices, leading to minimum cellular damage [179].

## Techniques and materials to deliver genetic payloads

As previously mentioned, genetic payloads are delivered into cells through vectors. Gene delivery systems are grouped into two categories: viral and non-viral vectors. In this section we will discuss advantages and disadvantages for both types of vectors, and their current and potential applications in the gene and cell therapy fields.

### Viral vectors

Viral vectors are delivery vehicles used to transduce human cells. In 1968, Rogers and Pfuderer were the first to perform proof of concept studies for virus mediated gene transfer using lysates of tobacco leaves infected with tobacco mosaic virus [180] (Table 3). The first instance of viral gene therapy performed on T cells ex vivo was conducted in 1990, on a four-year-old girl suffering from Severe Combined Immunodeficiency Defect (SCID). This treatment provided an encouraging signal to the field, even though the effects were temporary [181]. Despite the original success, a major setback occurred in 1999 when a patient died after receiving in vivo gene therapy [182]. The patient developed an intense inflammatory response against the adenovirus (AV) used as a vector, with blood-clotting followed by kidney, liver, and lung failure. Moreover, in another instance, 4 out of 10 patients with X-linked SCID treated with cell therapy developed vector-related T cell leukemia [183]. The transduction was performed ex vivo into autologous CD34^+^ hematopoietic cells, using a gammaretroviral vector. It’s worth noting that retroviral and lentiviral vectors have been used and monitored extensively in cell and gene therapy trials, and replication-competent retrovirus/lentivirus (RCR/L) and insertional oncogenesis related risks are considered unlikely to happen in T cell products [184] when FDA guideline criteria [185] are met.

**Table 3:** Milestones of gene and cell therapies, as well as notable technologies. Oligonucleotides therapies are not included in this table

Year	Notable technology	Gene therapy	Cell therapy
1962		Szybalski coined the term “gene therapy” [272–275]	
1968	Rogers and Pfuderer demonstrated virus mediated gene transfer [180]		
1972		Literature outlined the potential and concerns of gene therapy [276]	
1976	T cell growth factor interleukin 2 discovered [277]		
1982	EP system first described [204, 278]		
1983			Identification of human T cell antigen receptor [279–281]
1984	RVV transduced mammalian cells in vitro [282]		
1989	AV approved for clinical trial [283]		Concept of CAR-T appeared [284]
1990		First successful ex vivo gene therapy performed on an ADA-SCID patient [181]	
1993			1st gen CAR-T cells developed [285]
1996	1st gen LVVs created [286], ZFN gene editing system available [253]		
1997	SB transposon system designed for human cells [287]		FDA approved 1st anti-cancer monoclonal antibody Rituxan for NHL [288]
1999		A patient died due to immune response triggered by in vivo AV-based therapy [289]	
2000	4-component LNP reported [235]	Some SCID-X1 patients received ex vivo, RVV-based gene therapy [290]	
2003	First approved LVV use in a phase 1 trial (ex vivo, for HIV control) [291]	Reports of RVV related tumorigenesis in two patients after gene therapy for SCID-X1 [292]	
2005	New RNA modification to reduce immunogenicity developed [166], PB transposon system applied to mammalian cells [114]		
2006			TCR therapy applied to melanoma [293]
2007			First IND for CD19 CAR-T [294]
2009		Reports of eye disease treated in vivo with AAV-based vector [295]	
2010	TALEN gene editing system available [268]		CD19 CAR-T in NHL case reports [296, 297]
2011			CD19 CAR-T in CLL & ALL case reports [298–300]
2012	Crispr/Cas gene editing system available[259] [301]	EMA approved Glybera (withdrawn in 2017 for market reason) [302]	
2015			First BCMA CAR-T clinical trial [303]
2016	CRISPR/Cas9 applied to T cell engineering [304]	EMA approved Strimvelis [305]	
2017		FDA approved Luxturna [306]	FDA approved first cell therapies Yescarta [307]and Kymriah [308]
2018	Multiple CRISPR clinical trials combined CAR-T and PD-1 KO for cancer immunotherapies [304]		
2019		FDA approved Zolgensma [309] EMA approves Zynteglo [310]	
2020			FDA approved Tecartus [311]
2021			FDA approved Breyanzi [312] and Abecma [313]
2022			FDA approved Carvykti [314]

These severe adverse events represented a turning point toward the development of alternatives to viral vectors. However, over the years, research on viral vectors also led to significant improvements in their safety. More recently, CAR-T and gene therapy treatments based on viral vectors have been approved and commercialized (Table 4). A variety of viral vectors are currently available for clinical practice and several comprehensive reviews have been written about this topic [186–189].

**Table 4:** Details of the approved therapies based on viral vector technology

Trade name	Agency	First approval	First approved indication	Cargo	Delivery method	Admin	Pivotal clinical trial	Actual completion date if applicable
Glybera^®^	EMA	2012/10 (withdrawn 2017/10)	LPLD	LPL	AAV	in vivo	NCT00891306	2009/02—2011/04
Strimvelis^®^	EMA	2016/04	ADA-SCID	ADA	RVV	ex vivo	NCT00598481	2008/01—2019/06
Kymriah^®^	FDA	2017/08	r/r B-cell ALL	CD19 CAR	LVV	ex vivo	NCT02435849	2015/04—2020/01
Yescarta^®^	FDA	2017/10	r/r LBCL	CD19 CAR	RVV	ex vivo	NCT02348216	2015/04—2020/09
Luxturna^®^	FDA	2017/12	biallelic *RPE65* mutation-associated IRD	RPE65	AAV	in vivo	NCT00999609	2012/10—2015/07
Zolgensma ^®^	FDA	2019/05	SMA (type I)	SMN	AAV	in vivo	NCT03306277	2017/10—2019/11
Zynteglo™	EMA	2019/05 (withdrawn 2022/03)	TDT	HBB	LVV	ex vivo	NCT01745120	2013/08—2018/02
Tecartus™	FDA	2020/07	r/r MCL	CD19 CAR	RVV	ex vivo	NCT02601313	2015/11—2019/07
Breyanzi^®^	FDA	2021/02	r/r LBCL	CD19 CAR	LVV	ex vivo	NCT02631044	2016/01—2022/12
Abecma^®^	FDA	2021/03	r/r MM	BCMA CAR	LVV	ex vivo	NCT03361748	2017/12—2024/11
Carvykti ™	FDA	2022/02	r/r MM	BCMA CAR	LVV	ex vivo	NCT03548207	2018/06—2022/08

Despite successful developments, the main drawback for viral vectors remains their immunogenicity, especially in relation to inflammation [190, 191]. For example, there are significant challenges associated with using vectors based on adenovirus serotype 5 and adeno-associated virus type 2 in clinical settings. These viruses are so widespread that a significant number of people bear pre-existing immunity against them [192, 193]. Even repeated administration of viral vectors with low seroprevalence to patients may present serious challenges, due to risks in developing immunity against the vector and degeneration of the transduced tissue. Moreover, ectopic integration of viral DNA can also be responsible for insertional mutagenesis [194, 195]. This process can lead to disruption of tumor suppressor genes or activation of oncogenes, triggering neoplastic transformation of the host cells. Other disadvantages associated with viral vectors include their limited transgenic capacity, manufacturing challenges, and efforts related to scaling up the production process [196, 197]. Viral vector manufacturing presents technical barriers which may create a supply chain shortage and may slow the expansion of cell and gene therapy [198, 199]. Viral vectors also have a lengthy production process in terms of generating and testing the master and working cell banks [199, 200]*.* Continuous efforts are currently carried out to mitigate this issue. Due to these drawbacks, the development of non-viral vectors and delivery methods with lower immunogenicity remains a viable option.

### Non-viral vectors

Since the early 2000s, the use of non-viral vectors in clinical trials has increased steadily. This may be due to improvements in their efficiency, safety, stability of gene expression and specificity. Non-viral vectors have a lower testing burden and smaller storage footprint compared to viral vectors [201, 202]. Moreover, they are amenable for use in personalized therapies that target patients’ personal mutanome [202, 203]. These characteristics are important when deciding on a gene correction platform for treating one or a small number of patients.

**Fig. 1:**
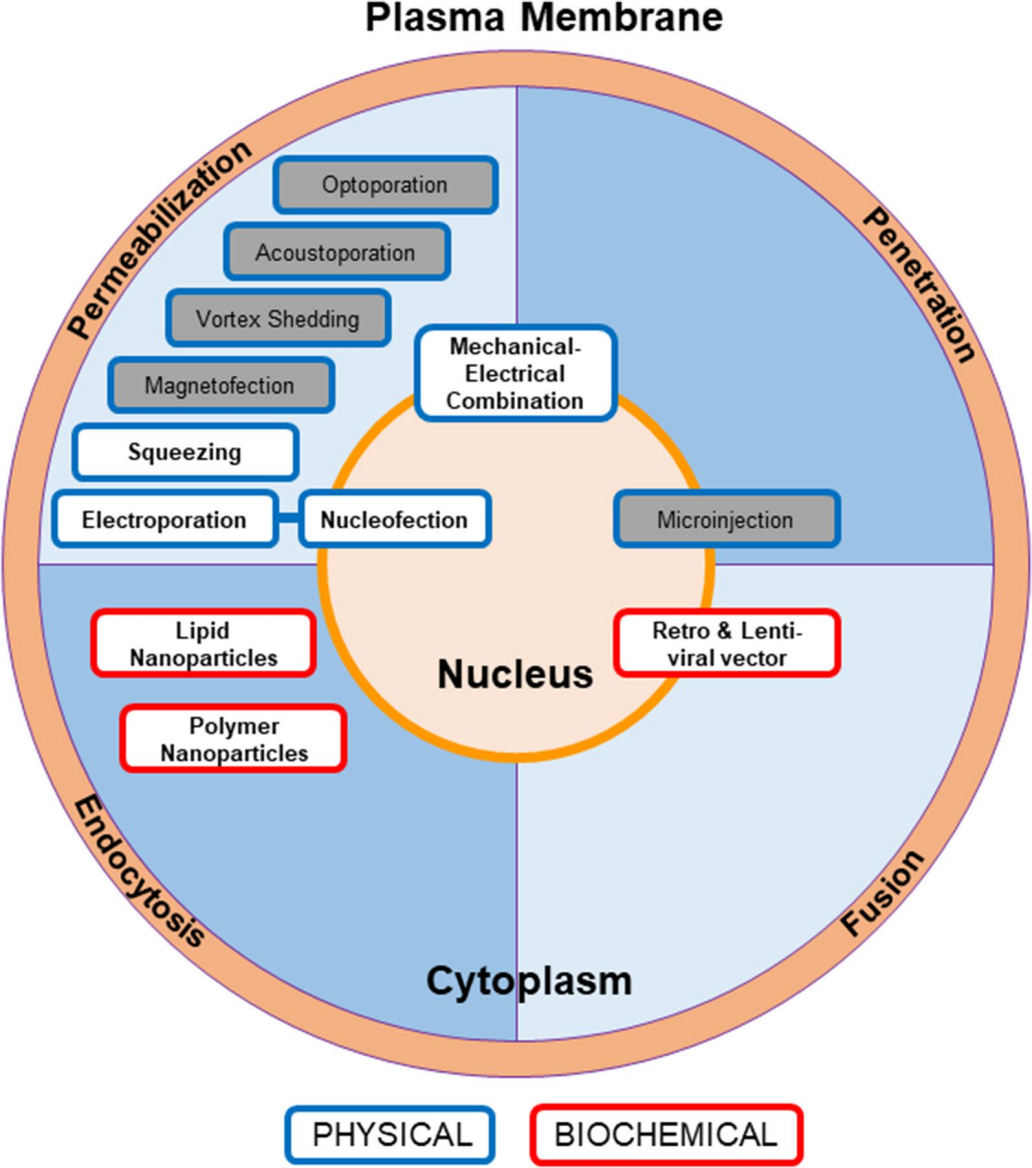
Common delivery methods are shown here with color coded boxes. The blue and red borders represent the physical or biochemical nature of the methods. Different delivery methods are positioned in color-coded sections to represent the mechanism of entry. The method with multiple mechanisms is positioned on the border of the mechanism involved. The methods not covered in detail in this review are grayed out. Specific references for each method can be found in the relative sections of the manuscript

Nevertheless, the optimal non-viral vector and delivery system still need to be tailored to the type of target cells and to the characteristics of the transgene, requiring a customized system. Commonly used non-viral vectors include transposons and episomes, both have been discussed above. Depending on its nature (DNA, mRNA, protein), the payload needs to pass through one or two barriers before reaching the genome: the cellular plasma membrane (the first barrier) and the nuclear membrane (the second barrier). Four different methods have been exploited so far to deliver the payload into the cells: fusion, penetration, permeabilization and endocytosis (Fig. 1). As mentioned above, viral transduction, due to its natural cell entry mechanism through fusion, has been utilized in commercialized cell and gene therapy products. In this section, we will focus on non-viral gene delivery, using physical or biochemical-based techniques. Physical methods utilize permeabilization and penetration to deliver a genetic payload into the cells, while biochemical approaches rely on endocytosis and fusion.

#### Physical delivery methods

Commonly used physical delivery methods include electroporation (EP) and microfluidic-based mechanical methods. These approaches create pores on the cellular membrane for gene entry via electric shock or mechanical stress. The payload is usually prepared in a specific buffer and does not require laborious preparation (e.g., envelope the payload in a viral capsid or coat with nanoparticles), significantly reducing the effort associated with this type of methodology. Physical methods do not have stringent limitations on payload size; however, as payload size increases, the need to obtain larger and more persistent pores may compromise cell viability. In bulk EP transfection, cells are mixed with the payload in a conductive buffer that is connected to two electrodes, then exposed to a brief electrical pulse for a few milliseconds [204, 205]. By combining different electrical voltages, pulse durations, and buffer chemistry, EP can be optimized to maximize transduction efficiency for different types of payloads and minimize cellular damage, even in hard-to-transduce cells, such as stem cells and T cells [206, 207]. Although the delivery efficiency is dependent on payload size, Zhao et al. [170] and Birkholz et al. [208], demonstrated that the efficiency of EP transfection can be up to 80% using pDNA, and higher than 90% in stimulated T cells transduced with mRNA [170, 208]. In another study, Distler et al. [209] designed an advanced EP technique called nucleofection which improves the transfection efficiency in unstimulated T cells, by using a unique combination of conductive buffer and electrical pulses [209].

Proteins can also be successfully delivered through EP. Due to its versatility in payload delivery, EP may be paired with new gene-editing techniques that require multiple components. In the first human clinical trial to assess the safety and feasibility of CRISPR-Cas9 gene editing of human T cells, the researchers used EP to deliver ~ 160 kDa ribonucleoproteins, targeting three genes in primary human T cells with the frequency of editing up to 45% in this trial [53]. KO efficiency could reach 85% to 98% in activated human T cells [42] and > 80% in other leukocytes [210]. CRISPR/cas9 system has been delivered with viral vectors such as lentiviral [211] and adenoviral vectors [212] and exhibited varied KO efficiencies, but few studies have directly compared efficiency of viral and non-viral methods with the same target and same CRISPR system. Multiple studies have also shown that EP is effective in delivering a transposon system into immune cells, with up to 65% transfection efficiency [213, 214]. EP-based transfection technologies have been developed to enable scalability, using different microfluidic approaches. For example, a semi-continuous flow and micro-fluidic EP devices have been developed to process large quantities of cells with high transfection efficiency for clinical applications [189].

EP transfection is also associated with several drawbacks. It requires special equipment and optimization for each cell type [215, 216] since fine tuning of EP conditions to achieve good viability and transfection efficiency is critical. EP conditions have been particularly difficult to optimize for certain cell types. To increase efficiency, high DNA concentration is generally used but can result in DNA toxicity in host cells. For bulk EP, non-uniform electric field distribution may cause Joule heating and bubble formation that could severely affect transfection efficiency and cell viability (reviewed in [217–219]). Recent developments could enhance the local electric field, thus lowering the operating voltage and preventing the formation of bubbles, therefore increasing cell viability up to 90% [220].

In addition to cell viability, multiple reports expressed concerns regarding the impact of EP on immune cells, as it may lead to alteration in gene expression, reduced expansion capacity and cytotoxicity [221–223]. To minimize the impact on primary immune cells, other physical methods have been explored, such as the squeezing method. By passing cells through a microfluidic device with constriction 30–80% smaller than the cell diameter, it is possible to create transient holes in the cell membrane. This method has successfully delivered protein, RNA, and DNA to multiple cell types, including embryonic stem cells and immune cells [224, 225]. It has been shown that the squeezing method has a minimal effect on gene expression and does not interfere with T cell activity, as observed with EP [224].

However, the application of squeezing technologies to human primary T cells still needs further study. Despite these recent advances, squeezing may not be suitable for in vivo gene therapy because target cells need to be isolated and processed ex vivo using specific equipment [226].

Recently, mechanical–electrical combination technology has been developed, combining nanostraw [227] or cell squeezing [228] and electric-field-driven transport. For the latter, cells are passed through microfluidic constrictions to disrupt the plasma membrane, then shocked with an electronic pulse to permeabilize the nuclear envelope. Nuclear delivery of pDNA was detected within 1-h post-treatment, and the integrity of the nuclear envelope was recovered within 15 min post-treatment. Cell viability is similar to the cells exposed to standard EP at the 24 h post-treatment time point [228].

Other mechanical methods commonly used in preclinical studies are fluid shear, vortex shedding, microinjections, acoustoporation, laser optoporation, and magnetofection (reviewed in [217, 229]).

#### Biochemical delivery methods

Nanoparticle chemistry has application to gene and drug delivery. Nanoparticle-based gene delivery, either lipid-based nanoparticles (LNPs) or polymeric nanoparticles (PNPs), are technologies centered on the encapsulation of the payload [230]. The cellular plasma membrane is negatively charged, which makes it difficult for negatively charged pDNA and mRNA to enter the cells by diffusion. Cationic nanoparticles bind to pDNA and mRNA to form lipoplexes and polyplexes that harbor a net positive charge and can enter the cell through endocytosis [231].

Lipid molecules have been used to transport genetic material into cells for a long time [232, 233]. Modern LNP systems appeared around the year 2000 to deliver DNA. They consist of four components: phospholipid, lipid-anchored polyethylene glycol (PEG), cholesterol, and ionizable lipid [234, 235]. The recent COVID-19 mRNA vaccines utilized this four-component LNP system demonstrating the efficiency and safety of delivering genetic material [236–238].

Their main drawback is the limited capacity to undergo endosomal escape, which affects the amount of genetic material that may reach the cytoplasm [239]. By incorporating the component that could target specific molecules, LNP delivery systems have the potential for precise in vivo delivery. This aspect was highlighted in a recent study using T cell-targeting LNPs to deliver mRNA encoding the CAR in vivo [172]. Overall, LNPs have low toxicity due to the natural and biological origin of the components and, under proper storage conditions, they may be stable for up to 150 days [240].

PNPs are another type of vehicle used for drug delivery, composed of natural carbohydrate polymers or synthetic polymers. Some PNP-based systems may exhibit higher stability and mechanical resistance compared to LNP-based systems [241–243]. The natural polymer chitosan (CS) is a cationic polysaccharide obtained from the exoskeleton of crustaceans such as crabs and shrimps. Chitosan nanoparticles can form electrostatic complexes with DNA, making it an attractive carrier for non-viral application [244]. It has been tested as a carrier for gene therapy in brain tumors [245], but it is also known to trigger an IL-1β response in a variety of cell types [246], which may be a concern for in vivo therapies.

Synthetic polymers present limited batch-to-batch variation. One example is polycationic polyethyleneimine (PEI) which is commonly used for gene delivery, thanks to its high transfection efficiency and high buffer capacity. Such a property is attributed to the proton sponge effect from the partially protonated amines on PEI chains (reviewed in [247]). PEI nanoparticles have been used to transfer large (12–14 kb) payloads, such as self-amplifying replicon RNAs (RepRNA) [248]. Recently, Olden et al. [249] developed an architecture of pDMAEMA polymers with 25% transfection efficiency for mRNA and 18% for pDNA in CD4^+^ and CD8^+^ primary T cells [249]. Another study achieved 12% transfection efficiency of pDNA in primary T cells [250].

Degradable synthetic polymers such as polylactide (PLA) and Poly (β-amino ester) (PBAE) have been developed to address concerns regarding the long-term toxicity of non-biodegradable polymers. PBAE exhibited robust transfection capabilities and efficient endosomal escape properties. It also showed promising results in cytosolic protein delivery in vitro and efficient CRISPR-Cas9 delivery in several cell types [251]. PNPs could also be manufactured in combination with ligands for in vivo tissue and cell targeting. A recent study indeed showed that PBAE nanocarriers successfully delivered CAR or TCR-encoding mRNA to circulating human primary T cells. Engineered T cells achieved tumor regression in xenograft mouse models [168].

## Conclusion

Multiple options are currently being explored to treat genetic disease using molecular tools, to restore gene expression to physiological conditions. As alternatives to viral vectors, non-viral vectors represent a potentially promising strategy due to comparable efficacy to viral vectors, for clinical applications.

In this review, we summarized the landscape of molecular tools, type of payloads, material and methods used as a strategy to regulate gene expression for gene and cell therapy. Knowledge about non-viral vectors is expanding exponentially and will likely prompt an acceleration in the application of new compounds in different medical specialties.

## Data Availability

Not applicable.

## Abbreviations

ADA-SCID: adenosine deaminase severe combined immunodeficiency
ALL: acute lymphocytic leukemia
ASP-siRNA: allele-specific siRNAs
AV: adenovirus
AAV: adeno-associated virus
bp: base pair
CRISPR: clustered regularly interspaced short palindromic repeats
Cas: CRISPR associated protein
CLL: chronic lymphocytic leukemia
DSB: double-strand break
EP: electroporation
EMA: European Medicines Agency
GCRs: gross chromosomal rearrangements
gRNA: guide RNA
IVT: in vitro-transcribed
LBCL: large B-cell lymphoma
LCC: linear covalently closed
LPLD: lipoprotein lipase deficiency
LNPs: lipid-based nanoparticles
LTR: long terminal repeat elements
LVV: lenti-viral vector
MAC, HAC: mammalian or human artificial chromosomes
MCL: mantle cell lymphoma
MNs: meganucleses
MM: multiple myeloma
NHL: non-Hodgkin's lymphoma
NHEJ: Non-Homologous End Joining
dCas: nuclease dead Cas
PB: PiggyBac
PBAE: poly β-amino ester
PEI: polycationic polyethyleneimine
PEG: polyethylene glycol
PLA: polylactide
PNPs: polymeric nanoparticles
PTGS: post-transcriptional gene silencing
PD-1: programmed cell Death protein 1
RepRNA: replicon RNAs
RNAi: RNA interference
RISC: RNA-Induced Silencing Complex
RITS: RNA-induced transcriptional silencing
r/r: relapsed/refractory
RVV: retro-viral vector
SA: sandwich transposon
S/MAR: scaffold/matrix attachment region sequences
SCID: Severe Combined Immunodeficiency Defect
shRNA: short hairpin RNA
SSTR: single-stranded template repair
SB: Sleeping Beauty
siRNA: small interfering RNA
SMA 1: spinal muscular atrophy type I
TDT: transfusion-dependent beta-thalassemia
TIRs: terminal inverted repeats
TALENs: transcription activator-like effector nuclease
TEs: transposable elements
FDA: U.S. Food and Drug Administration
ZFNs: zinc finger nucleases
